# Supplementary material for: Cardiac kinematic parameters computed from video of in situ beating heart
Source: Sci Rep. 2017 Apr 11;7:46143. doi: 10.1038/srep46143 (PMC5387404; doi:10.1038/srep46143)
Supplement: Supplementary Information [file srep46143-s1.pdf]

Cardiac kinematic parameters computed from video of *in situ* beating heart

Lorenzo Fassina, Ph.D.[1,2,+], Giacomo Rozzi, M.Sc.[3,+], Stefano Rossi, Ph.D.[3], Simone Scacchi, Ph.D.[4], Maricla Galetti, Ph.D.[5], Francesco Paolo Lo Muzio, B.Sc.[3], Fabrizio Del Bianco, M.Sc.[1,2], Piero Colli Franzone, Ph.D.[6], Giuseppe Petrilli, M.D.[7], Giuseppe Faggian, M.D.[7] and Michele Miragoli, Ph.D.[3,8,9*]

[1]Dipartimento di Ingegneria Industriale e dell'Informazione, Università degli Studi di Pavia, Via Ferrata 1, 27100 Pavia, Italy

[2]Centre for Health Technologies (C.H.T.), Università degli Studi di Pavia, Via Ferrata 1, 27100 Pavia, Italy

[3]Dipartimento di Medicina e Chirurgia, Università degli Studi di Parma, Via Gramsci 14, 43124 Parma, Italy

[4]Dipartimento di Matematica, Università degli Studi di Milano, Via Saldini 50, 20133 Milano, Italy

[5]CERT, Centro di Eccellenza per la Ricerca Tossicologica, INAIL-exISPESL, Università degli Studi di Parma, Via Gramsci 14, 43124 Parma, Italy

[6]Dipartimento di Matematica, Università degli Studi di Pavia, Via Ferrata 1, 27100 Pavia, Italy

[7]Dipartimento di Cardiochirurgia, Università degli Studi di Verona, Ospedale Borgo Trento, P.le Stefani 1, 37126 Verona, Italy

[8]Humanitas Clinical and Research Center, Via Manzoni 56, 20090 Rozzano, Italy

[9]Institute of Genetic and Biomedical Research, National Research Council, Via Manzoni 56, 20090 Rozzano, Italy

[+]These authors contributed equally to this work

Supplementary Information

This section includes:

Supplementary Methods

Supplementary Figure legends

Supplementary Video legends

Supplementary References

Supplementary Figures

Supplementary Methods

Experimental animals

The animals were kept in single-sex groups of four individuals from weaning (4 weeks after birth) until the onset of the experiments, in a temperature-controlled room at 20-24°C, with the light on between 7:00 AM and 7:00 PM. The bedding of the cages consisted of wood shavings; food and water were freely available. Animals were anesthetized with a mixture of ketamine chloride 40 mg/kg ip (Imalgene, Merial, Milano, Italy) and medetomidine hydrochloride 0.15 mg/kg ip (Domitor, Pfizer Italia S.r.l., Latina, Italy) for the *in vivo* experiments; all efforts were made to minimize suffering. We used nine male rats in total. Six male rats were enrolled in i) the experiments related to the cardiac force measurement with a given mass (n = 6); ii) the same animals were used for the ischemia/reperfusion experiments (n = 6) and iii) three of them included in the “reproducibility” experiments, monitoring parameters every ten minutes for 1 h before ischemia/reperfusion (n = 3). Three male rats were included in mechanically-induced AV block experiments. This study

was carried out in accordance with the recommendations in the “Guide for the Care and Use of Laboratory Animals” of the Italian National Institute of Health. The protocol was approved by the Veterinary Animal Care and Use Committee of the University of Parma and conformed to the National Ethical Guidelines of the Italian Ministry of Health (Protocol # 41/2009 and 59/2012).

Parameters calibration for the mathematical model of ischemia simulation

The intra (i)- and extra (e)- cellular conductivity coefficients of the Bidomain model, along (l) and across (t) the fiber direction, were $\sigma^i_l = 3$, $\sigma^e_l = 2$, $\sigma^i_t = 0.31525$, $\sigma^e_t = 1.3514$, all in $m\Omega^{-1}cm^{-1}$. The parameters of the strain energy function were the same of the original paper[1] except for the bulk modulus, which amounted to 200 kPa. The size of the ischemic region was 1 cm × 1 cm, developing along the whole transmural thickness, from the endocardial to the epicardial surface. From the bioelectrical point of view, ischemic conditions were modeled modifying the following parameters of the ten Tusscher model: the extracellular potassium concentration was increased from 5.4 mM to 8 mM; the maximal conductance of the I_{Na} and I_{CaL} currents was decreased of 25%; the parameters of the I_{KATP} current were modified as in[2] for ischemia stage 2. From the mechanical point of view, ischemic conditions were modeled by reducing the active tension and passive tissue stiffness of 10% and 25% with respect to their normal values, respectively. The excitation process was initiated by stimulating three endocardial anterior apical sites and one endocardial posterior apical site, mimicking an idealized Purkinje network. Two simulations were performed, one for a healthy tissue (HEALTH) and one for a tissue with a transmural ischemic region (ISCH). In both cases, three beats were simulated, at basic cycle length of 500 ms.

Supplementary Figure legends

Supplementary Figure 1

Velocities in the x direction obtained at different marker radius. We used a video marker radius equal to 20, 15, or 10 pixel (px) for **a**, **b** and **c** respectively. We found that a radius equal to 15 pixel was the minimum required to correctly follow the ventricle's tissue and, then, to identify the cardiac beats. n = 3

Supplementary Figure 2

Marker's trajectories and the related velocity vectors obtained at different acquisition rates. The rate of 500 fps **(a)** gave the best trajectory details, the rates of 250 **(b)**, 125 **(c)**, 100 **(d)**, and 50 **(e)** could be considered acceptable, whereas the other lower rates (≤25 fps in **f**) were significantly affected by the aliasing phenomenon with loss of trajectory details and were excluded from this study.

Supplementary Figure 3

Reproducibility of the acquired data in three different rat hearts. (a) Maximum velocity module (pixel/s) monitored every 10 min for 1 h at 1000 fps. **(b)** Same as (a) for kinetic energy (pixel^2/s^2). **(c)** Same as (a) for acceleration module (pixel/s^2). **(d)** Same as (a) for maximum displacement module (pixel). Marker radius = 60 pixel. n = 3.

Supplementary Figure 4

Evaluation of the kinetic energy during the periodic motion of a pendulum. (a) First acquired image of the pendulum at the highest point. Scale bar = 1 cm. **(b)** Trajectories and accelerations during five seconds of recording (with a maximum inaccuracy of three pixels in the y direction). **(c)** Coordinate x vs. time. **(d)** Kinetic energy at the highest point (ca. zero, E_k min), at the intermediate point (E_k int, $p<0.05$) and at the lowest point (maximum kinetic

energy, E_k max, $p<0.001$). The statistics was calculated with one-way ANOVA. Data are showed as mean ± SEM. n = 10.

Supplementary Figure 5

Numerical simulation results of epicardial transmembrane (V) and extracellular (UE) potential distributions. (a) V distribution at t = 120 ms after the onset of stimulation in the healthy tissue (HEALTH). **(b)** V distribution at t = 120 ms after the onset of stimulation in the tissue with transmural ischemia (ISCH). **(c)** UE distribution at t = 120 ms after the onset of stimulation in the healthy tissue. **(d)** UE distribution at t = 120 ms after the onset of stimulation in the tissue with transmural ischemia. Numbers at the bottom of each panel represent the minimum value, the potential step and the maximum value respectively.

Supplementary Figure 6

Functional parameters of human beating hearts. (a) Mean ejection fraction (EF%, left, **$p<0.01$) and mean fractional shortening (FS%, right, $p>0.05$) of seven patients before and after CABG. **(b)** Acceleration module, kinetic energy, maximum velocity module and maximum displacement module before (white) and after (black) CABG ($p<0.05$ only for the acceleration module; the p-value was calculated from Student's t-test, normal distribution was checked by means of the Kolmogorov-Smirnov test).

Supplementary Figure 7

Kinetic energy related to the Frank-Starling effect following an AV block. **(a)** Video cardiogram (ViCG) for the marker displacement of spontaneously beating rat heart with an AV block between the third and the fourth beat; red arrow: mechanically induced AV block (20 kPa of air pressure delivered for 1 s). **(b)** Related kinetic energy measured for each

cardiac cycle. Black dots: single beat kinetic energy measured for each cycle. Red dot: single beat kinetic energy after AV block. **(c)** Same as (a) for spontaneously beating human heart (patient #1). Red arrow: spontaneous AV block. **(d)** Related kinetic energy measured for each human heartbeat. Red dot: single beat kinetic energy after AV block.

Supplementary Video legends

Supplementary Video 1

Example of tracking for the video marker positioned onto the epicardial surface and related trajectories.

Supplementary Video 2

Ten-seconds recording of spontaneously beating rat heart with a known mass firmly connected onto the epicardial surface.

Supplementary Video 3

Example of how an air-pressure flow, delivered on pulmonary cone, induced AV block on rat heart.

Supplementary References

1 Eriksson, T. S. E., Prassl, A. J., Plank, G. & Holzapfel, G. A. Influence of myocardial fiber/sheet orientations on left ventricular mechanical contraction. *Math Mech Solids* **18**, 592-606, doi:10.1177/1081286513485779 (2013).

2 Rodriguez, B. *et al.* Effect of acute global ischemia on the upper limit of vulnerability: a simulation study. *American journal of physiology. Heart and circulatory physiology* **286**, H2078-2088, doi:10.1152/ajpheart.01175.2003 (2004).

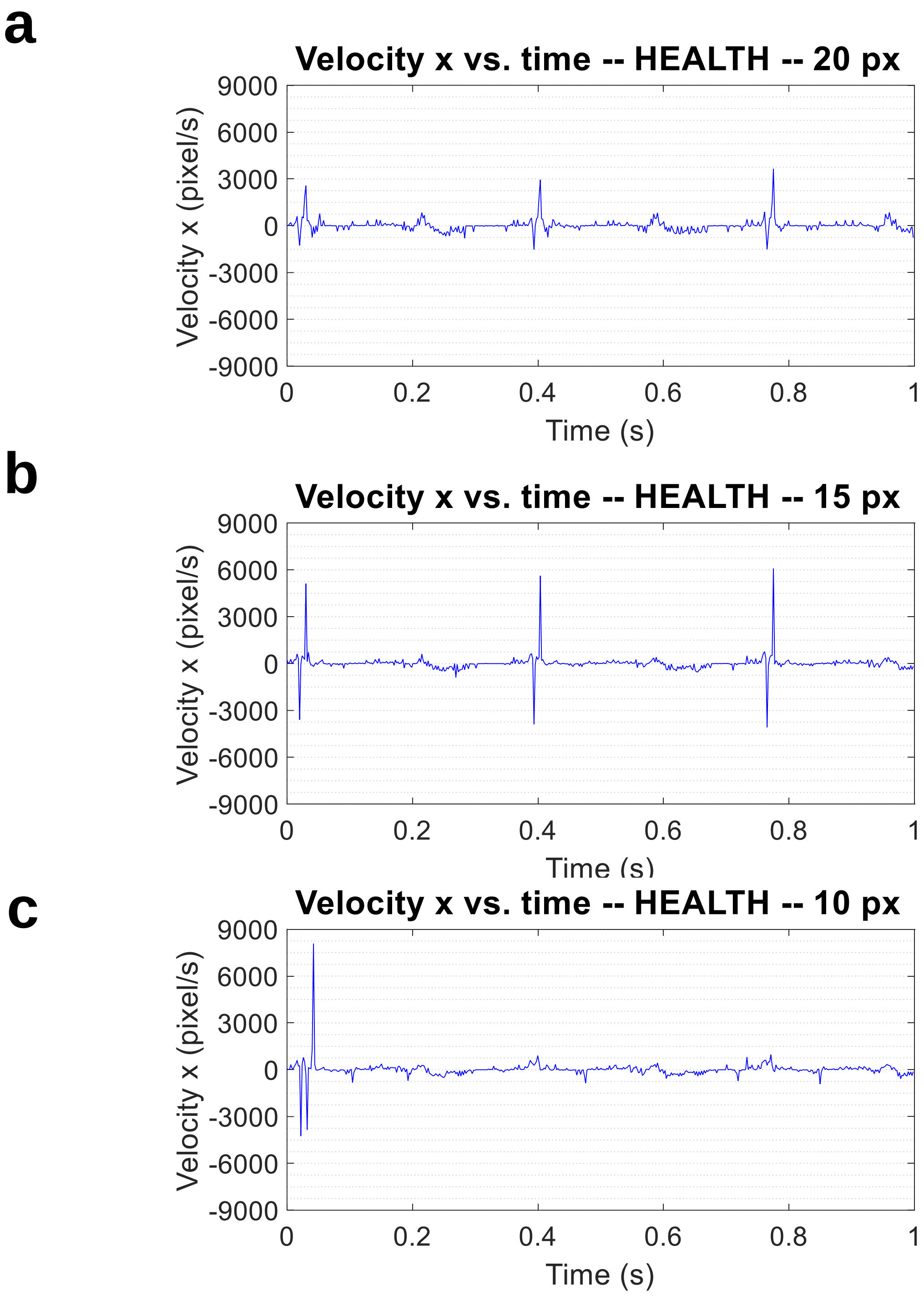

Supplementary Fig. 1

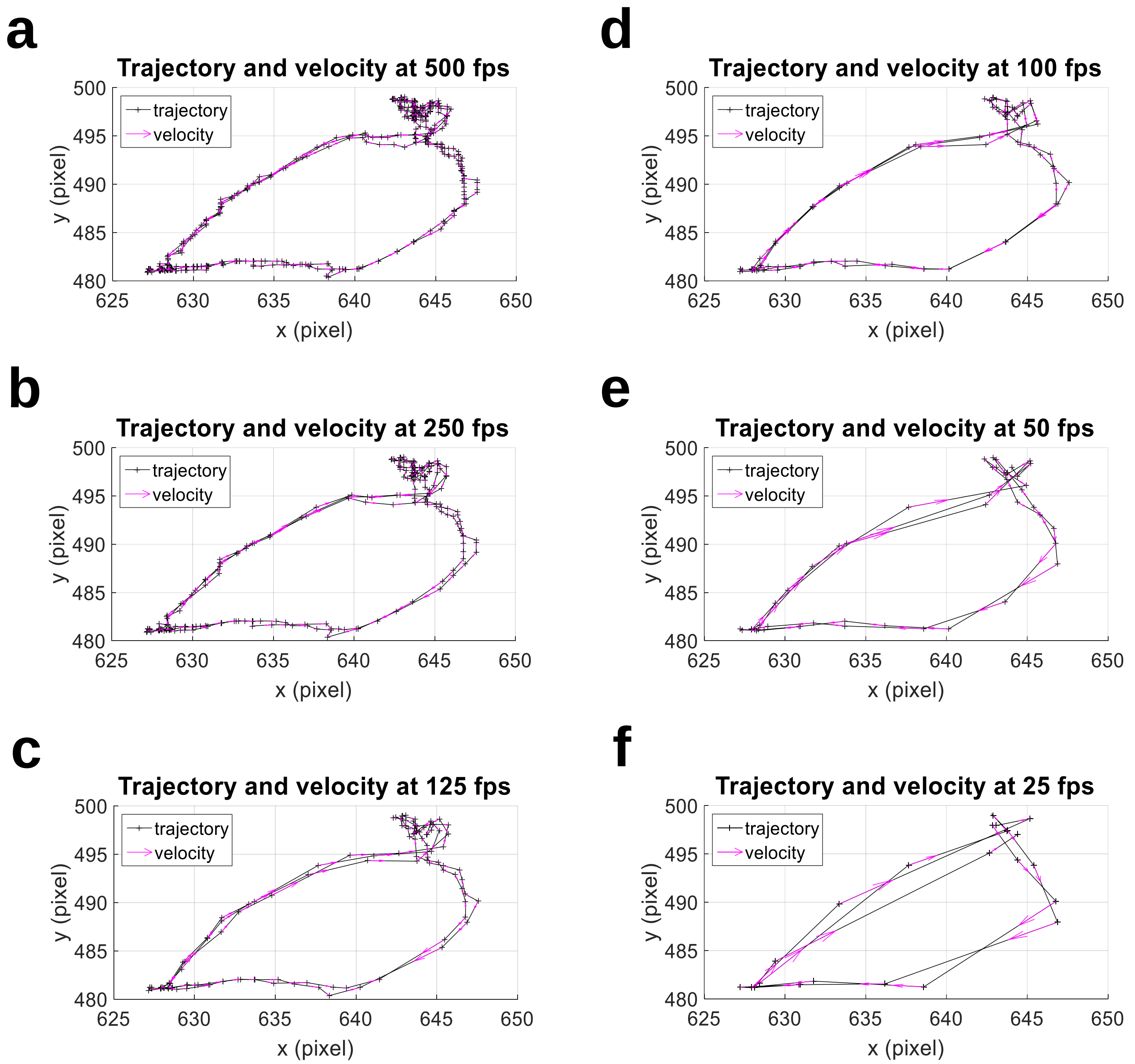

Supplementary Fig. 2

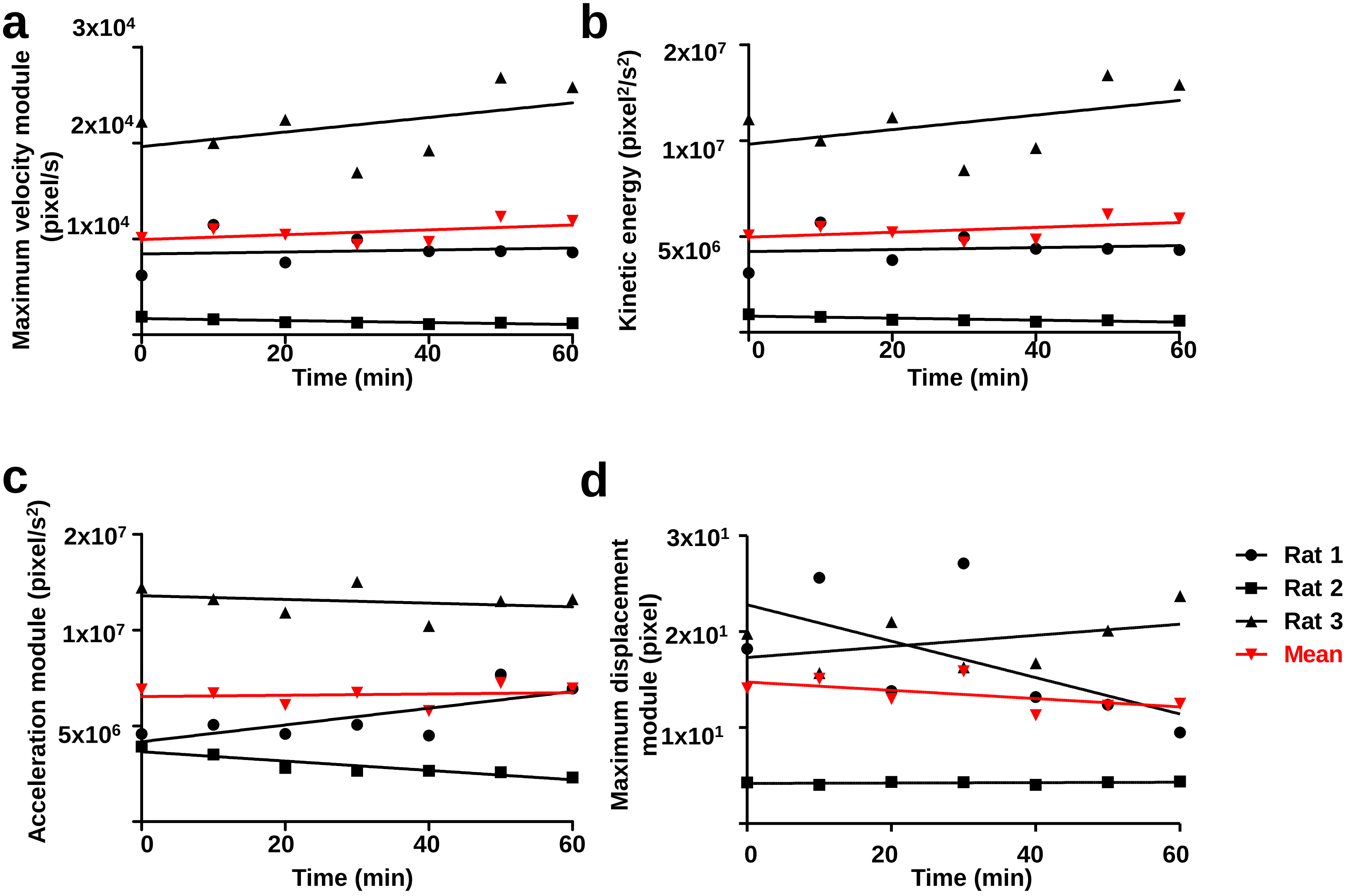

Supplementary Fig. 3

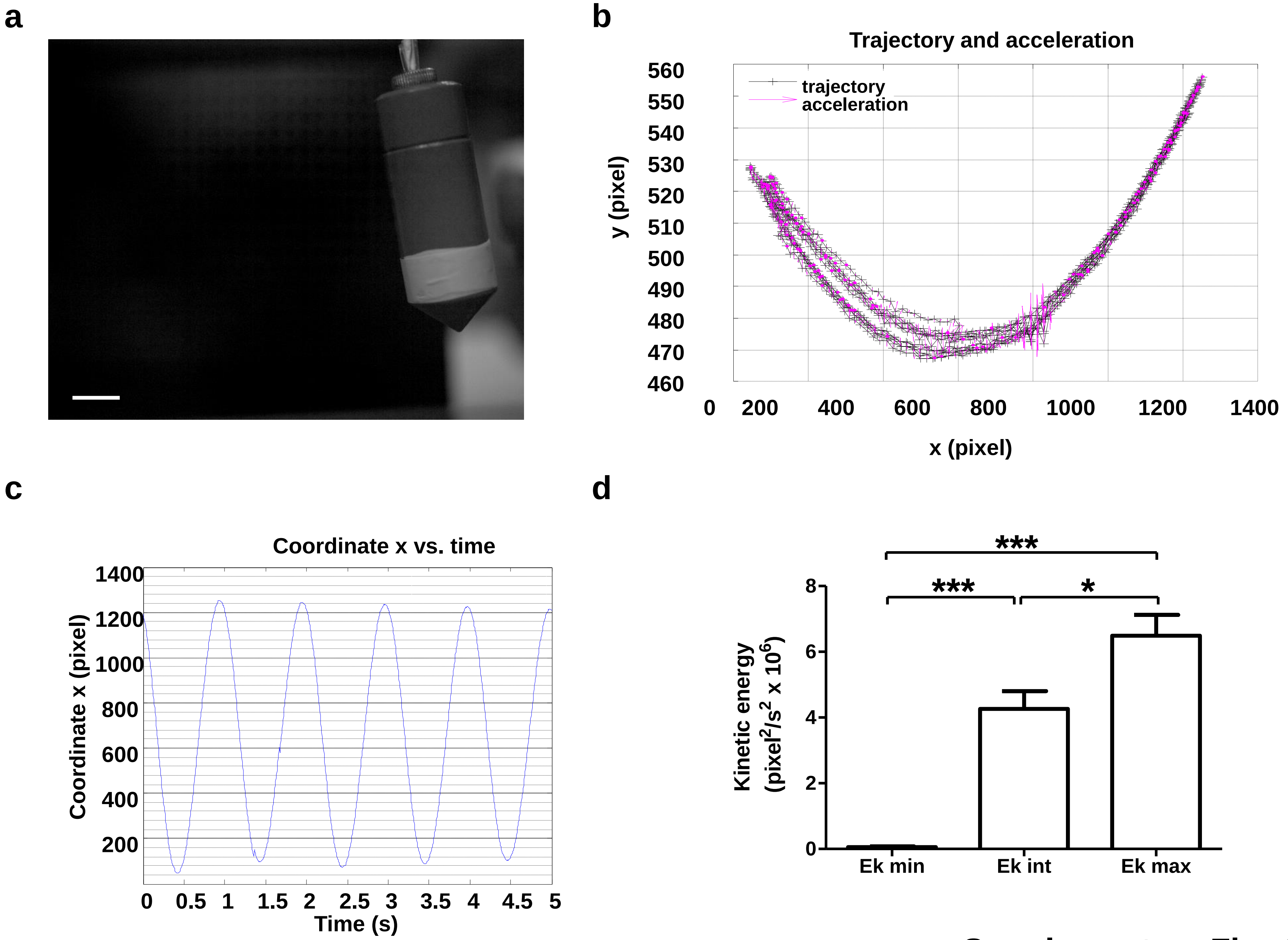

Supplementary Fig. 4

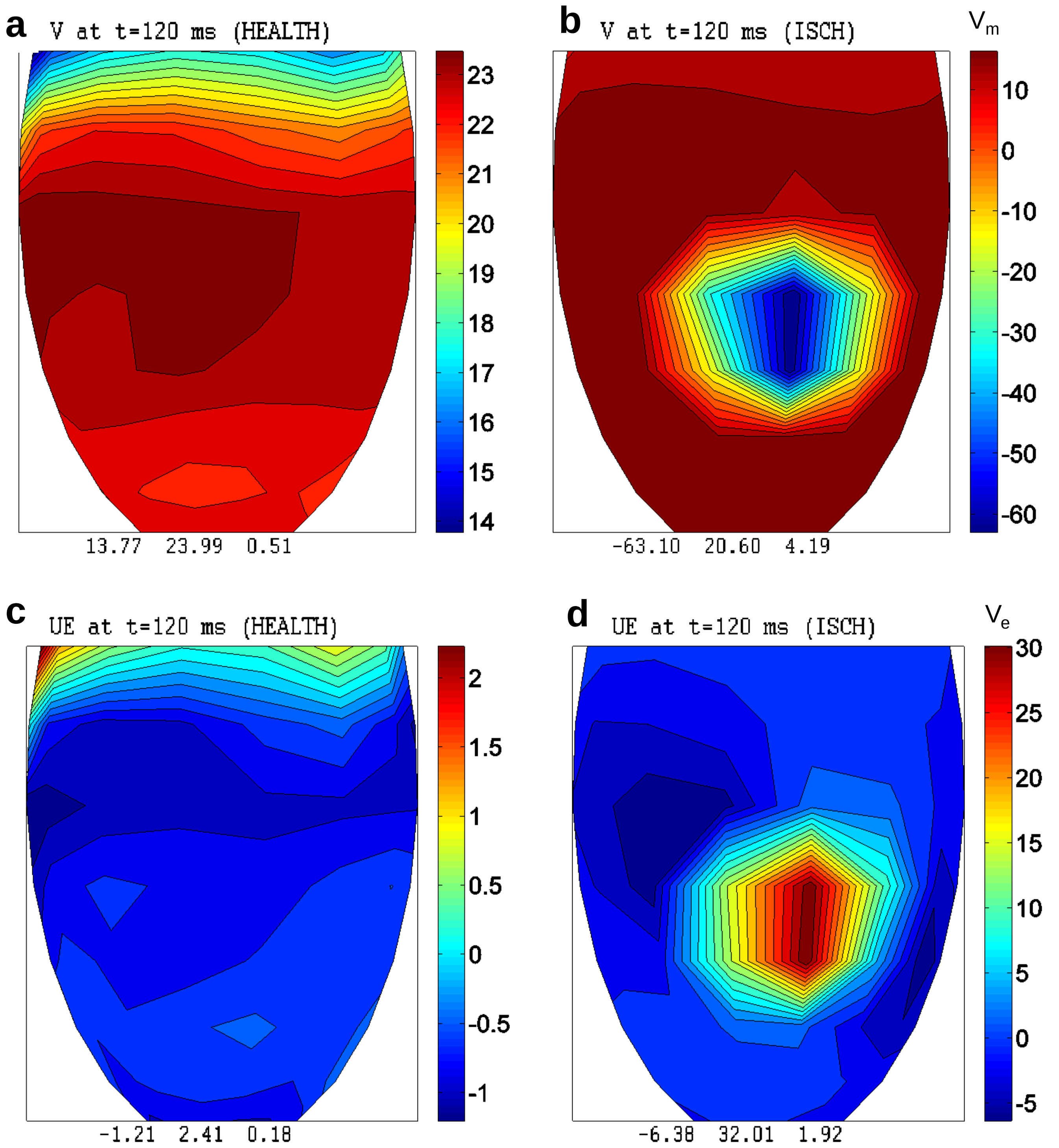

Supplementary Fig. 5

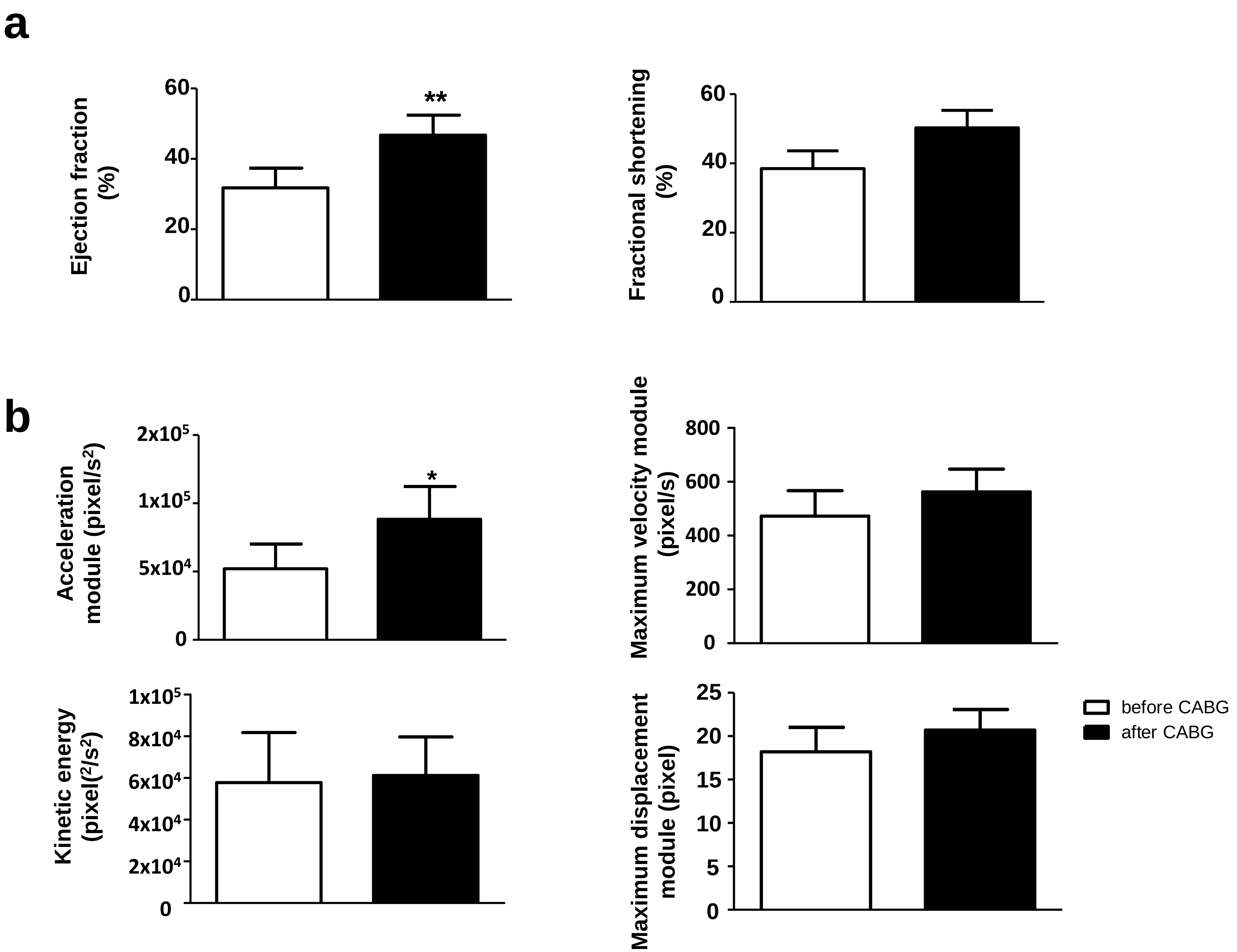

Supplementary Fig. 6

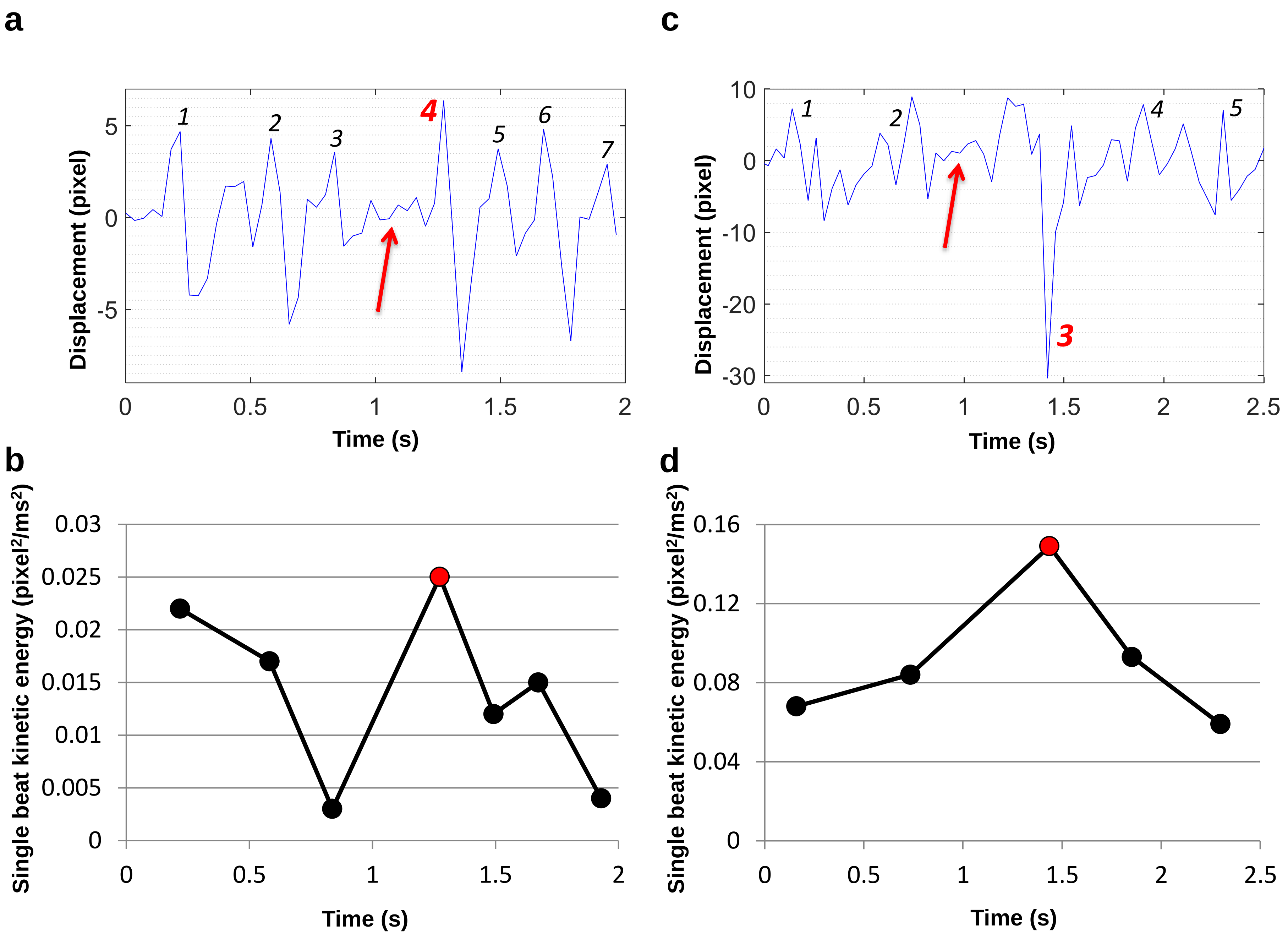

Supplementary Fig. 7
